# Unifying Virulence Evaluation in *Toxoplasma gondii*: A Timely Task

**DOI:** 10.3389/fcimb.2022.868727

**Published:** 2022-04-28

**Authors:** Rafael Calero-Bernal, Mercedes Fernández-Escobar, Frank Katzer, Chunlei Su, Luis Miguel Ortega-Mora

**Affiliations:** ^1^ SALUVET, Animal Health Department, Faculty of Veterinary Sciences, Complutense University of Madrid, Madrid, Spain; ^2^ Disease Control Department, Moredun Research Institute, Edinburgh, United Kingdom; ^3^ Department of Microbiology, University of Tennessee, Knoxville, TN, United States

**Keywords:** *Toxoplasma gondii*, virulence, phenotype, harmonization, lethal parameters, non-lethal parameters

## Abstract

*Toxoplasma gondii*, a major zoonotic pathogen, possess a significant genetic and phenotypic diversity that have been proposed to be responsible for the variation in clinical outcomes, mainly related to reproductive failure and ocular and neurological signs. Different *T. gondii* haplogroups showed strong phenotypic differences in laboratory mouse infections, which provide a suitable model for mimicking acute and chronic infections. In addition, it has been observed that degrees of virulence might be related to the physiological status of the host and its genetic background. Currently, mortality rate (lethality) in outbred laboratory mice is the most significant phenotypic marker, which has been well defined for the three archetypal clonal types (I, II and III) of *T. gondii*; nevertheless, such a trait seems to be insufficient to discriminate between different degrees of virulence of field isolates. Many other non-lethal parameters, observed both in *in vivo* and *in vitro* experimental models, have been suggested as highly informative, yielding promising discriminatory power. Although intra-genotype variations have been observed in phenotypic characteristics, there is no clear picture of the phenotypes circulating worldwide; therefore, a global overview of *T. gondii* strain mortality in mice is presented here. Molecular characterization has been normalized to some extent, but this is not the case for the phenotypic characterization and definition of virulence. The present paper proposes a baseline (minimum required information) for the phenotypic characterization of *T. gondii* virulence and intends to highlight the needs for consistent methods when a panel of *T. gondii* isolates is evaluated for virulence.

## Introduction

*Toxoplasma gondii* is an apicomplexan parasite, globally distributed, with a heteroxenous life cycle that virtually comprises all homoeothermic animals, including humans, as intermediate hosts and felids as definitive hosts. The zoonotic, abortifacient and foodborne nature of the parasite makes toxoplasmosis a relevant public and animal health concern worldwide. Although the disease course is normally asymptomatic, immunocompromised and pregnant hosts are important risk groups. Clinical toxoplasmosis is mostly due to tachyzoite invasion and proliferation in different cells of the host, with subsequent destruction and necrotization of the infected tissues.

*Toxoplasma gondii* possesses a significant genetic and phenotypic diversity that have been proposed to be partly responsible for the variation in clinical presentations. Similar to genetic markers, parasite strain specific differences could be defined by “phenotypic markers” (Dardé et al., 2020). In the context of *T. gondii*, phenotype is related to virulence and lethality in laboratory mice, which is well defined for the three archetypal clonal types of the organism. *Toxoplasma gondii* type I (belonging to haplogroup 1) strains have been traditionally classified as highly virulent (100% cumulative mortality, LD_100_ = 1), type II (belonging to haplogroup 2) strains are considered of intermediate virulence (99-30%, LD_50_ ≥ 10^3^), and type III (belonging to haplogroup 3) strains are defined as non-virulent (< 30%, LD_50_ > 10^5^) (Sibley and Boothroyd et al., 1992; Su et al., 2002; Dardé et al., 2020). Likewise, most of the South American divergent strains (haplogroups 4 - 10) have been characterized as highly virulent using virulence in mice as a phenotypic marker (Grigg and Suzuki, 2003; Khan et al., 2007).

Quantitative trait locus (QTL) mapping analyses of virulence in mice of a F1 progeny, derived from sexual recombination experiments of representative strains of the three *T. gondii* archetypal genotypes (I×II, I×III and II×III crosses), resulted in the identification of some members of a family of serine/threonine protein kinases, found in rhoptries, as key determinants of acute virulence in mice (Saeij et al., 2006; Taylor et al., 2006; Behnke et al., 2011; Reese et al., 2011; Behnke et al., 2015). The *ROP18*, *ROP5*, and *ROP16* genes encode three polymorphic rhoptry protein kinases that in a different but synergic manner contribute to the evasion of host immune response controlling the accumulation of interferon-γ induced immunity-related GTPases (IRGs) on parasitophorous vacuole (PV) membranes and subsequent parasite destruction (Behnke et al., 2012; Niedelman et al., 2012). The proven role played by these effectors motivated the interest to develop molecular typing markers based on their sequences to quickly infer the degree of virulence of *T. gondii* strains. Subsequent studies concluded that the allelic combination of *ROP18/ROP5* is highly predictive of virulence in mice across globally distributed *T. gondii* strains (Dubey et al., 2014; Shwab et al., 2016). Nonetheless, there is growing evidence that this correlation is inconsistent for some genotypes (Bernstein et al., 2021; Fernández-Escobar et al., 2021).

In practice, parasite virulence assessment follows a simplistic and host-centred criterion, based mainly on the pathogenicity in mice. In most cases this is reduced to the calculation of mortality rate. A much more comprehensive definition of the virulence of *T. gondii* strains should combine the study of infection effects on the hosts (*e.g.*, mortality, morbidity, immune responses dynamics) and other parameters of the parasite’s own fitness, such as success in transmission (*e.g.*, cystogenesis, oocyst production rate) or the rate of asexual multiplication (*e.g.*, invasion and proliferation rate) (Poulin and Combes, 1999).

*Toxoplasma gondii* genetic characterization methodologies have been largely standardized in the past two decades. However, phenotypic characterization procedures have not been subject to the same criticism and standardization due to its complexity. When it comes to phenotypic characterization of strains, many aspects, including environmental factors, host species, genetics of a given host species, and parasite stage can influence the outcome of the infection (Mukhopadhyay et al., 2020). There is wide evidence that long-term laboratory conditions (*i.e.*, regular passages in cell culture or mice) can determine the biological behaviour of the parasite (Khan et al., 2009; Saraf et al., 2017; Sánchez-Sánchez et al., 2019). In addition, different hosts can have completely different infection outcomes even if challenged with the same isolate, and different infection routes can also affect the infection consequences (Oliveira et al., 2016; Yang et al., 2017; Taniguchi et al., 2018; Hassan et al., 2019; Sánchez-Sánchez et al., 2019). There was an attempt to standardize the calculation of cumulative mortality rate by Saraf et al. (2017), accepted as a consensus for virulence in mice assessment, but the fact is that this protocol has been applied in very few publications (Costa Viegas de Lima et al., 2019; Fernández-Escobar et al., 2020; Uzelac et al., 2020; Fernández-Escobar et al., 2021). Current animal welfare policies, which strictly frame scientific research, aim to minimize the use of laboratory animals and to refine the experiments. In the published scientific literature, there is a lack of concordance in the biological parameters measured as well as in the experimental conditions such as doses, infection routes or duration of the study. Consequently, it is difficult to derive general conclusions and to make comparisons of virulence of isolates presented in different studies, and it becomes evident that an in-depth review of methodologies is needed.

## *In Vivo* Models for Virulence Assessment: State and Limitations

Animal models have been widely used to characterize *T. gondii* virulence and host responses to the infection by this parasite. Small rodents are natural hosts of *T. gondii* and likely play a major role as intermediate host for transmission of *T. gondii* infection. Most reported studies used laboratory mice as animal models due to their relative ease for handling and management. Domestic animals such as sheep, pig and chickens were also used mainly to assess host responses to parasite infection.

### Current Normalized *In Vivo* Mouse Method Based on Mortality Ratio

Until now, mortality in mice has been considered the main parameter for the virulence evaluation of *T. gondii* strains, and it was established as the ratio between casualties and the number of infected animals challenged with the strain under study. Different subspecies of mouse have been used for *T. gondii* infection *in vivo* modelling, such as Swiss Webster, CD-1, C57BL/6, BALB/c or Kunming strains, among many others (Wang et al., 2013a; Taniguchi et al., 2018; Fukumoto et al., 2020; Uzelac et al., 2020). *Toxoplasma gondii* infection has been widely studied using cell-culture derived tachyzoites (or zoites grown in the mouse peritoneal cavity) that are intraperitoneally (IP) or subcutaneously (SC) inoculated into naïve laboratory mice (Howe et al., 1996). Despite not constituting a natural infection route, this model has the advantages of reproducibility, ease of inoculation, and accurate administration of challenge dose (Sibley et al., 1999). However, variants of this procedure using other parasite stages have been also implemented, including IP injection of bradyzoites contained in tissue cysts (Taniguchi et al., 2018; Gatkowska et al., 2019), or *per os* (PO) inoculation of tissue cysts (McLeod et al., 1984; Khan et al., 2007; Taniguchi et al., 2018; Arcon et al., 2021) and oocysts (Yang et al., 2017; Chiebao et al., 2021). These variants are inherently less reproducible due to the variable bradyzoite content of a given tissue cyst, or the difficulties in guaranteeing the oral dosage, as well as less feasible due to the complexity of oocysts production; but on the other hand, oocyst- or bradyzoite-induced infections are much more representative of what occurs in nature (Sibley et al., 1999). Consistent with the *T. gondii* life cycle, oocyst-mediated infections are known to be more pathogenic than bradyzoite- and finally, tachyzoite-induced infections (Dubey et al., 1977; Dubey et al., 1981; Dubey, 2006; Saraf et al., 2017). Logically, the doses tested (number of parasites inoculated) vary greatly between experimental designs depending on the parasite life cycle stage used, but they also do so between experiments that use the same parasite stage. All these mentioned factors (the route of inoculation, the parasite stage, the dose, and the host species or even subspecies) along with the number of passages in mice or cell culture, have been demonstrated to drastically affect the degree of parasite virulence (Saraf et al., 2017). Currently, there is a greater awareness that it is important to keep passages of evaluated isolates low before they become lab adapted, but the use of strains maintained for a long time under laboratory conditions (“laboratory strains”) or for which the passage number is not known still remains widespread in literature (Khan et al., 2014). Therefore, despite the numerous studies published on virulence evaluation (**Supplementary Table S1**), the large diversity of conditions and methodologies implemented, with heterogeneous interpretations and fragmented data, makes it difficult to draw conclusions about the real *T. gondii* population structure in terms of virulence.

After initial attempts for normalization (Su et al., 2002; Taylor et al., 2006), the only available standard operating procedure for the evaluation of cumulative mortality rates was recently published (Saraf et al., 2017). According to the authors, cumulative mortality rate calculation implies the use of outbred mice (*e.g.*, Swiss Webster [SW] or CD-1 mouse strains), at least three consecutive doses of IP inoculated tachyzoites, and the recording of casualties among those successfully infected animals by day 28 post-inoculation (dpi). It should be pointed out that in some contexts, animal welfare regulations prevent getting ethical approval when mouse assays include several inoculation groups. In most publications, mortality rate in mice is the only parameter evaluated, which implies an overly simplistic and narrow view of virulence. Until Saraf’s publication, there was no consensus in the literature about how the mortality rate in mice should be calculated, so it is frequent to find lethality estimations/assumptions based on animal casualties during isolation procedures (Pena et al., 2006; Clementino Andrade et al., 2013; Shwab et al., 2016; Vilares et al., 2017), and even valuable attempts have been made to quantify the parasites in the inoculum (Mercier et al., 2010). Despite being a substantial attempt to standardize the procedure, some limitations should be pointed out. A parameter originally valued in the strain’s virulence assessment was the median lethal dose (LD_50_; Probit tests: https://probitanalysis.wordpress.com/2016/07/07/first-blog-post/; Finney, 1971; Shittu et al., 2020), especially since the discovery of absolute lethal doses (LD_100_) of a single parasite in the case of the strains related to haplogroup 1 (Sibley and Boothroyd, 1992; Su et al., 2002; Salman et al., 2021). However, this calculation has fallen out of favour, similarly to duration of survival post infection analyses (Wang et al., 2013a; Costa Viegas de Lima et al., 2019). Accurately determined, these parameters could offer new insights into dose-dependency and infection dynamics.

### Phenotypic Diversity of the Global *Toxoplasma gondii* Population

**Figure 1:**
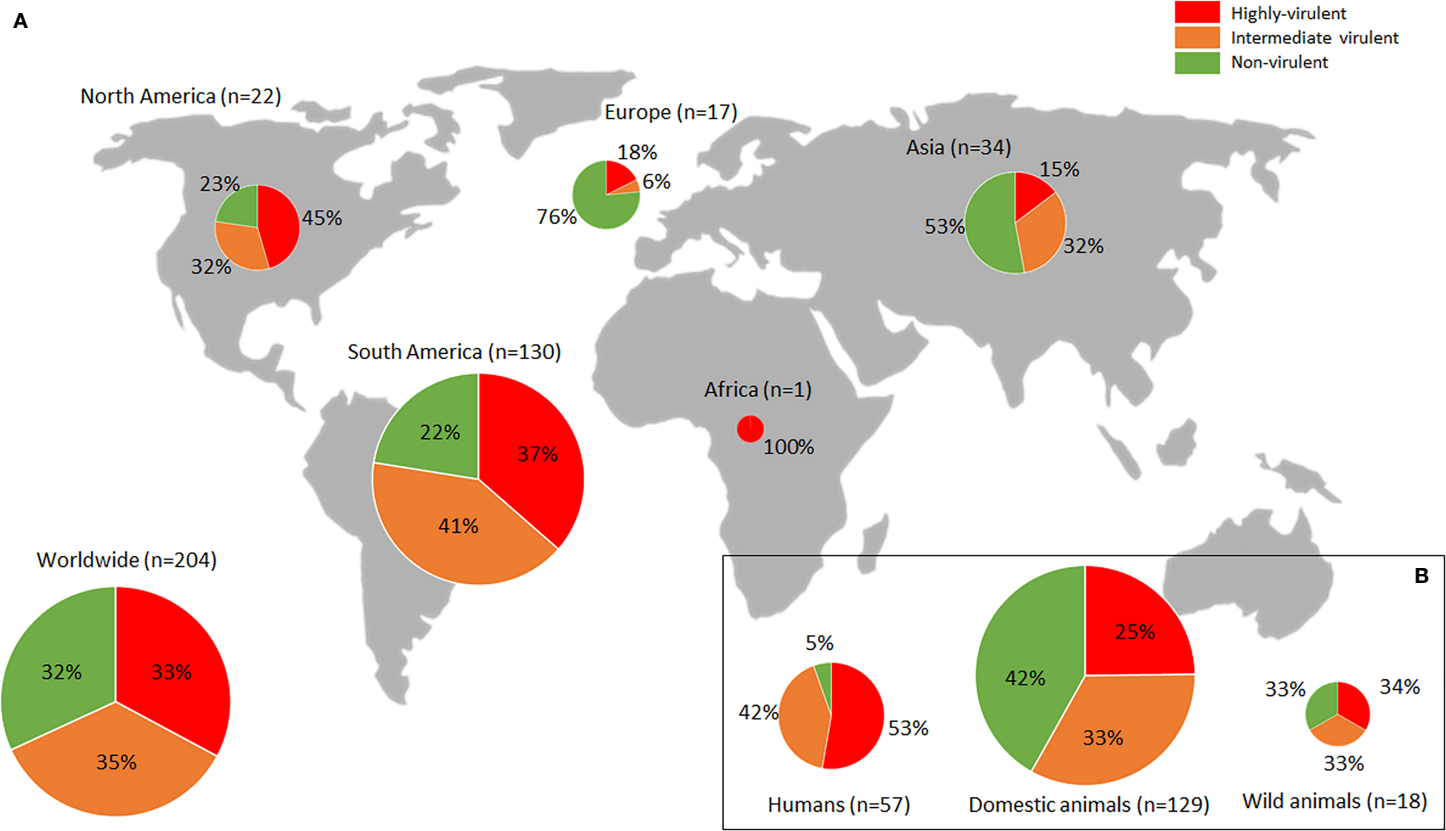
Worldwide *Toxoplasma gondii* phenotypic diversity distribution. **(A)** Proportion of highly-, intermediate- and non-virulent isolates (based on mortality rates in mice) found in each continent. **(B)** Figures observed pertaining to infected hosts (humans, domestic or wild animals). Sizes of pie charts correlate with total number of isolates (n). Only studies in which mortality in outbred mice was assessed, implementing at least 3 doses of IP or SC inoculated tachyzoites (serial 10-fold dilutions from 1 to 10^6^ parasites/mouse), and 28-dpi animal monitoring (Saraf et al., 2017) were considered. Data used are compiled in **Supplementary Table S1**.

The assessment of virulence in mice for a large number of *T. gondii* strains worldwide is contained in literature. The present section aims to critically examine the available phenotypic data, defined as mortality in mice, from *T. gondii* isolates worldwide, trying to provide an overview of the virulence profiles of *T. gondii* populations in the different continents. The PubMed database (https://pubmed.ncbi.nlm.nih.gov/) was searched combining the terms “Toxoplasma gondii”, “virulence characterization” and “pathogenicity”; 2644 investigations published until December 2021 were found. Only *T. gondii* isolate virulence studies involving assays measuring tachyzoite stage induced mortality in mice were considered, while studies focusing on *in vitro* assays or other parasite stages (oocysts or bradyzoites) were excluded. Infections based on these other stages account for only a minority of investigations, which is further limited by methodological variations (see previous section). Other rodent *in vivo* models (*e.g.*, rats) were not included. Data from laboratory strains (*e.g.*, RH, GT1, CTG, ME49, PRU, VEG) were not covered (except when obtained shortly after strain isolation), in order to better describe the real phenotypic diversity of the *T. gondii* populations globally. In total 62 studies were selected, involving 311 isolates (see **Supplementary Table S1**). Isolate IDs, host of origin, available genetic (*e.g.*, ToxoDB#, ROP18/ROP5 alleles) and geographical (*e.g.*, country, continent) features, experimental conditions for mouse mortality assessment, and other evaluated parameters, were extracted for each reference. Due to the heterogeneity of data presentation, mortality rate was re-calculated in each reference (when possible) based on Saraf et al. (2017) criteria (analysis of three sequential inoculation dosages, with the lowest dose resulting in only partial infection of the animals), and isolates were classified into “Highly virulent” (100% mortality rate), “Intermediate virulent” (99–30%) and “Non-virulent” (<30%) categories according to Su et al. (2002). Calculations considering 4 or more doses when available were also included. In the **Figure 1**, only studies in which mortality in outbred mice was assessed implementing at least 3 doses of IP or SC inoculated tachyzoites (serial 10-fold dilutions from 1 to 10^6^ parasites/mouse), and 28-dpi animal monitoring were filtered (Saraf et al., 2017) (33 studies; 204 isolates), in order to map the *T. gondii* virulence diversity more accurately by increasing methodological homogeneity. Data is geographically biased; it is worth noting the sizable proportion of South American (including Caribbean) isolates (n=130) compared to the scarcity of those from Europe (n=17), North America (n=22) or Asia (n=34) and the extremely poor representation of the African continent (n=1). Generally, the *T. gondii* population in South America is mostly considered highly virulent, in association with a notably diverse, endemic genetic structure (Shwab et al., 2014; Shwad et al., 2018). However, present data show a proportion of South American highly virulent isolates that are not more common than those found in North America (37% *vs.* 45%) (**Figure 1A**). In both regions, almost one in four strains proved to be non-virulent (22-23%). In contrast, European isolates are broadly considered non-virulent, in relation with the prevalent genetic clonality found in the continent (involving mainly genotypes II and III) (Shwab et al., 2014; Shwab et al., 2018). Nonetheless, present data showed a not insignificant percentage of highly virulent strains in Europe (18%) (**Figure 1A**). Interestingly, the global/worldwide *T. gondii* virulence in mice distribution displays a certain balance between highly-, intermediate- and non-virulent strains (33%, 35% and 32%, respectively) (**Figure 1A**). Collected virulence data distribution within the different hosts of origin is also strongly biased; the vast majority of isolates assessed were obtained from domestic animals (n=129/204), while only 18 were isolated from wild animals (**Figure 1B** and **Figure S1**). Ignoring this fact, phenotypic data extracted showed an almost equal proportion of the different virulence degrees in strains infecting animals. In detail, isolates from domestic animals seem to be slightly less virulent than isolates from wildlife, which could correspond with the apparent more virulent character of “wild” strains *vs.* “domestic” strains described in North America (Jiang et al., 2018). Data fromhuman isolates (n=57/204) is partially biased as most of them came from clinical cases, which could explain that only 5% of the strains evaluated showed a non-virulent profile, while more than a half presented a highly virulent character (**Figure 1B**). Nevertheless, although a certain phenotype is probably being selected in isolates of human origin, it should be pointed out that there is no clear link between virulence in mice and in humans. On the other hand, researchers deal with an extra issue when it comes to human isolates due to the uncertainty of the geographical origin of some infections, with importation or migration related cases, only solvable by comprehensive epidemiological surveys.

Present data collection probably provides a view of *T. gondii* worldwide population phenotypic diversity more realistic than previously described (Shwab et al., 2016; Shwab et al., 2018) based mainly on mortality rates calculated during the isolation process. The bioassay of digested tissue samples in mice cannot be considered as a method for virulence evaluation due to the lack of parasite quantification or control of inoculum composition, the assessment of a single “dose” and the widespread use of a very low numbers of mice (typically 3). Nonetheless, it should be noted that since standardized models are relatively expensive assays as they involve the use of a higher number of experimental animals (quite restrictive and regularized), it is common for the strains subjected to these experiments to have been pre-selected due to their more disparate behavior, which may result in an extra bias specially in underrepresented continents such as Europe. Data extracted from standardized mouse models (**Supplementary Table S1**) identified 40.1% of highly virulent strains in the southern hemisphere, while this accounted for 25.3% in the northern hemisphere. This strongly contrasts with figures described in the Americas by Shwab et al. (2018), where 61% of 427 isolates from South/Central America were categorized as highly virulent in mice, while only 7% of 193 isolates from North America were found to be virulent.

### Other *In Vivo* Experimental Models

Little is known about how parasite virulence in mice extrapolates to other relevant hosts. Since the first characterization studies of *T. gondii* strains, there has always been an interest in associating virulence in mice with an outcome in human clinical infections (Sibley and Boothroyd, 1992). However, there are only a few comparative studies available, which indicate that drastically different infection outcomes occur in different hosts challenged with the same isolate (Taniguchi et al., 2018; Hassan et al., 2019; Sánchez-Sánchez et al., 2019; Xia et al., 2020).

#### Rodents

Efforts have been made to investigate if other rodents can be used as animal models to characterize virulence in *T. gondii*. In contrast to laboratory mice, which are highly susceptible to toxoplasmosis (Zenner et al., 1998), rats have been repeatedly demonstrated to be resistant to acute infection, remaining asymptomatic or even resistant to chronic infection by preventing tissue cysts formation in the case of some rat breeds (Sergent et al., 2005; Cavaillès et al., 2006). Therefore, it is considered that the laboratory rat infection model represents a better system for studying the immune resistance of humans to *T. gondii* infection than the mouse model (Loeuillet et al., 2019). In an experiment carried out in the USA, chronic toxoplasmosis was induced in Sprague Dawley female rats after oral inoculation with oocysts of 11 *T. gondii* strains of seven different genotypes. After 60 days, distribution, location and size of tissue cysts and pathological lesions in their brains were assessed by immunohistochemistry to investigate whether the parasite genotype could affect these virulence parameters (Dubey et al., 2016). Interesting differences between strains were found and some aspects of the parasite infection dynamics in rats were clarified. In a French study, Lewis (LEW) and Fischer (F344) rats were inoculated intraperitoneally with 10^7^ tachyzoites of the GUY008-ABE (haplotype 5, ToxoDB # unknown) or the Prugniaud (PRU; haplotype 2, ToxoDB #3) strains; weight loss, survival time, parasite dissemination and histological lesions were assessed. Resistant LEW and susceptible congenic LEW.BN.c10-F rat infections were also carried out to study the number of brain cysts developed by each strain 2 months after inoculation. Complementarily, parasite proliferation was evaluated *in vitro* in primary rat peritoneal macrophages. Final findings in this model demonstrated the hypervirulent phenotype of the South American (French Guiana) GUY008-ABE strain in contrast to the avirulent profile of PRU laboratory strain (Loeuillet et al., 2019). Recently, guinea pigs were also proposed as a suitable model for human congenital toxoplasmosis in experimental infections of pregnant guinea pigs being administered 10, 100 or 500 oocysts of *T. gondii* strain ME49 at different time points during gestation (Grochow et al., 2021). The impact of the dose, the duration of infection and the gestational stage at infection on the seroconversion, survival rate of dams, fate of the offspring, parasite loads in various offspring tissues and organs and the integrity of the brains of the offspring were assessed. This model, together with those developed in pregnant mice (Liu et al., 2013; Müller et al., 2017; Sánchez-Sánchez et al., 2019), are examples of how pregnant rodent models are considered good options to study human congenital toxoplasmosis due to the haemochorial placentation that primates (including humans) and rodents have in common.

#### Sheep

Among domestic animals, sheep are sensitive to toxoplasmosis. Thanks to the development of standardized experimental sheep infection models, the knowledge of the pathogenesis of ovine toxoplasmosis has increased considerably in recent years (Benavides et al., 2011; Castaño et al., 2014; Castaño et al., 2016; Benavides et al., 2017). A recent study (Sánchez-Sánchez et al., 2019) compared the *in vivo* phenotype of a recently obtained type II isolate (TgShSp1; ToxoDB #3) with the type II reference ME49 (ToxoDB #1) in pregnant and non-pregnant mice, as well as in pregnant sheep. Although the *in vivo* non-pregnant mouse infections and complementary *in vitro* assays indicated that the laboratory isolate ME49 was clearly more virulent than TgShSp1, there were no differences between these two isolates for fetal/lamb mortality, lesions, or number of *T. gondii*-positive lambs when pregnant ewes were challenged with oocysts. Reviewing the literature revealed that virulence assessments in sheep models are scarce.

#### Pig

Pigs are also considered sensitive to toxoplasmosis. Although several experimental attempts to reproduce congenital toxoplasmosis in pigs have been reported, these have not been consistently successful (Moller et al., 1970; Dubey et al., 1990; Jungersen et al., 2001; Basso et al., 2015; Basso et al., 2017). The pathogenicity of different *T. gondii* strains of diverse host origin was compared after intravenous (IV) inoculation of 10^4^ tachyzoites in 7-week-old pigs (Jungersen et al., 1999), assessing parameters such as rectal temperature, weight loss, histopathological lesions in several organs, IgG and IgM antibody levels, haptoglobin and TNF-α serum levels, among others. Later, an experimental infection in pregnant minipigs inoculated intravenously with 3 × 10^4^ tachyzoites of different strains (Jungersen et al., 2001) showed marked differences in acute illness, associated abortions, and evidence of the parasite in the gilts or their foetuses. BR-1 mini pigs infection was proposed as a suitable model for human toxoplasmosis (Miranda et al., 2015), with groups of animals intramuscularly inoculated with 10^7^ RH tachyzoites or orally infected with 660 ME49 tissue cysts, where clinical signs, parasitaemia, parasite burden in diverse organs, histopathological lesions, haematology or serum biochemistry, among other parameters, were evaluated. In another study (Taniguchi et al., 2018) micro minipigs were dosed orally with 900 tissue cysts of the Japanese isolate TgCatJpGi1/TaJ, previously classified as type III (ToxoDB# not provided), resulting in no clinical signs of infection. When tachyzoites of the same isolate were IP inoculated into laboratory mice no clinical signs of infection were observed either but 100-80% lethality was found in mice orally inoculated with low doses (100, 50 and 10) of tissue cysts of this isolate. Furthermore, a recently obtained Japanese isolate (TgCatJpOk4) showed notable mortality (60%) and morbidity (80%) rates when micro minipigs were IP inoculated with 10^7^ tachyzoites (Taniguchi et al., 2019), which is in concordance with its previously demonstrated 100% mortality in mice (Fukumoto et al., 2020). In an interesting comparative study (Xia et al., 2020), the virulence of a type PRU (ToxoDB #3) Chinese isolate (TgPIG-WH1) obtained from an aborted piglet was assessed in mice and pigs. TgPIG-WH1 was less virulent than the RH and ME49 reference strains in mice (35%, 100% and 80% mortality rate, respectively), but showed a strong pathogenicity in pigs with higher mortality, more severe pathological lesions, and higher IgG levels in serum in comparison to infections with the ME49 strain. Overall, *T. gondii* virulence assessments in pig models are not standardized, and comparison of results from different studies is difficult.

#### Chicken

Apart from experimental infections of sheep or pigs, other *in vivo* models could be found in the literature. Although chickens are considered resistant to clinical toxoplasmosis, and only a few reports of clinical toxoplasmosis are available worldwide, the fact that chickens are one of the most important meat resources for humans and the high seropositivity rates found in some areas, justifies an interest in developing infection models for this species (Dubey, 2021). Experimental infections of 7 to 28-day-old Broiler chickens by IP injection with 10^8^ tachyzoites of the mouse-virulent RH and JS strains were conducted (Wang et al., 2014). Clinical signs, survival time, parasite detection in pooled tissues and histopathological lesions were evaluated. The mortality rate in 7-day-old chickens infected with the JS strain (100%) was higher than with the RH strain (70%), but the infections did not produce relevant clinical manifestations in the rest of the challenged animals, and similar results were found for the other parameters evaluated for both strains.

## Complementary Approaches on the Determination of Virulence in *Toxoplasma gondii* Field Strains

### Non-Lethal Parameters

Current animal welfare policies aim to minimize the use of laboratory animals, replace them when possible, and to refine the experiments to reduce animal suffering. In this regard, important improvements can be introduced for the evaluation of virulence using the mouse model by assessing additional non-lethal parameters involved in virulence (**Table 1**). A much more comprehensive view of the virulence of *T. gondii* strains should combine the study of infection effects on the host (*e.g.*, lethality and tissue lesions) with other aspects inherent to the parasite’s own fitness. The oocyst production rate is a direct indicator of the parasite’s transmission success, and it has been studied in several publications in the past (Dubey et al., 2002; Dubey et al., 2003). The demonstrated loss of the capacity to produce oocysts, as a consequence of successive passages in cell culture or mice, is considered as a loss of virulence (Frenkel et al., 1976; Lindsay et al., 1991; Dubey et al., 1999). In particular, a study of oocyst shedding in domestic cats of several genetically diverse *T. gondii* strains from French Guiana showed difference of fecundity among them, which may potentially affect their transmission (Khan et al., 2014). However, the use of cat models has long been controversial and currently is not accepted by the wide scientific community, in ethical terms. Another useful way to measure parasite transmissibility is to evaluate parasite cystogenesis, the capacity and capability of *T. gondii* to form tissue cysts. The central nervous system (CNS) is the tissue *par excellence* to study the development of cysts during the chronic phase of the infection but the *T. gondii* tropism towards immune privileged organs also involves ocular or muscle tissues (Jiang et al., 2020; Yang et al., 2020; Dubey, 2021). Cysts can be quantified and even measured (diameter) by microscopic observation of fresh/unstained (Dubey et al., 2012) and immunostained fixed brain sections or brain homogenates (Masatani et al., 2020; Wang and Sibley, 2020). An indirect way to quantify the presence of cysts in the mouse brain is to measure the parasite burden in the CNS from 3 weeks pi by quantitative PCR (Fernández-Escobar et al., 2020; Fernández-Escobar et al., 2021; Salman et al., 2021). A literature review (Watson and Davis, 2019) compiled and described *T. gondii* experimental latent infections in murine models for the quantification of brain cysts, in order to find key factors on data variance and to propose optimized protocols; however, the conclusions were not as informative as expected because of the fragmentation of data gathered. Some non-archetypical strains showed decreased potential to develop into bradyzoites *in vitro*, bradyzoites with decreased resistance to pepsin treatment, and formation of a lower number and smaller tissue cysts, associated with a limited oral transmission (Fux et al., 2007).

**Table 1:** Summary of non-lethal parameters for the assessment of *in vivo* virulence of *Toxoplasma gondii* strains (ordered by reliability and informativeness).

Parameters evaluated	Sample to be tested	Method of evaluation	Information gathered/represented	Key references
**Parasite load in tissues**	Both parenchymatous and non- parenchymatous organs (brain-chronic phase, lungs-acute phase)	qPCR	Number of parasites/mg of tissue	Hill and Su, 2012; Fernández-Escobar et al., 2021; Salman et al., 2021
**Cystogenic capacity**	Parenchymatous organs (brain)	Immunostaining	Number of tissue cysts/field-section	Masatani et al., 2020
		Direct counting in brain homogenates	Number of tissue cysts/mg of tissue or by whole organ	Wang and Sibley, 2020
**Morbidity and survival time**	None	Animal monitoring by daily direct observation with clinical signs scoring*	Cumulative morbidity rate calculation, survival time	Fernández-Escobar et al., 2020
**Histological lesions**	Both parenchymatous and non-parenchymatous organs (brain and striated muscle-chonic phase, lungs-acute phase)	H&E staining, IHC, light microscopy	Frequency and severity of the lesions Presence/absence of parasites. Scoring needs to be implemented	Chiebao et al., 2021; Fernández-Escobar et al., 2021
**Cytokines expression**	Tissues (spleen, mesenteric lymph nodes)	mRNA expression by RT-qPCR	Cytokines profile (Fold change; Ct values)	Chiebao et al., 2021
	Serum	ELISA	Serum level	Araujo and Slifer, 2003
**Haptoglobin levels in acute phase**	Serum	Electroimmunoassay; ELISA	Serum level	Jensen et al., 1998; Jungersen et al., 1999; Jungersen et al., 2002
**Antibody levels (IgG)**	Serum, plasma and whole blood	ELISA, IFAT, MAT	Serum level	Yang et al., 2021
**Behavioural changes**	None	Image monitoring	Scoring needed	Bezerra et al., 2019

*Humane endpoints need to be set. IHC, immunohistochemistry.

As a reflection of virulence *in vivo*, parasite burdens in different organs have been determined after short times post-inoculation in many studies. Apart from CNS, parasite burdens in lung, spleen, kidney, liver, ocular tissues, mesenteric lymph nodes, diaphragm or even blood has been also studied in literature (Zenner et al., 1998; Djurković-Djaković et al., 2012; Hill and Su, 2012; Hill et al., 2012; Wang et al., 2013b; Hamilton et al., 2019; Fernández-Escobar et al., 2020; Fernández-Escobar et al., 2021). Real-time PCR was used to monitor the distribution of *T. gondii* in different murine tissues during the infection with 10^2^ or 10^6^ tachyzoites (IP) of the RH strain, or 10 cysts (PO) of the ME49 strain (Djurković-Djaković et al., 2012); this study concluded that the level of parasite burden in the lungs seemed to be critical for mice survival/parasite virulence. This result agrees perfectly with what was observed previously (Loeuillet et al., 2019), where pneumomegaly, and the greater parasitic load and tissue destruction in the lungs were strongly associated with the hypervirulence of the non-canonical GUY008-ABE isolate (haplogroup 5, ToxoDB # unknown). On the other hand, some studies pointed out ocular tropism as a virulence related trait, with a more frequent parasite DNA detection in ocular tissues from mice infected with more virulent strains (Hamilton et al., 2019; Chiebao et al., 2021; Fernández-Escobar et al., 2021). These findings are in agreement with data from human ocular toxoplasmosis in Brazil, where lesions in the retina are the most common clinical manifestation (Dubey et al., 2012). From a practical point, all these parasite distribution assays could be carried out in parallel to mortality rate evaluation, by selecting a dose of interest for tropism evaluation (Fernández-Escobar et al., 2021). Histological and immunohistochemical examinations for describing pathological lesions in tissues after *T. gondii* infection have been carried out in several studies, and even though they are considered essential to demonstrate the definitive cause-effect in clinical diagnostic reports, for virulence assessment this technique has less discriminatory power (Yang et al., 2017; Hamilton et al., 2019; Fernández-Escobar et al., 2020; Chiebao et al., 2021).

Another interesting approach to assess differences in virulence of *T. gondii* strains is to study differential expression of cytokines and other markers of the immune response during infection. The levels of mRNA expression in spleen and mesenteric lymph nodes of IFNγ, IL-12, T-cells surface markers CD8, CD4 and CD25, as well as the receptor adapter MyD88 and the chemokine receptor CXCR3, were evaluated and compared during mice infection with the archetypal non-virulent M4 strain (type II variant, ToxoDB #3), a non-archetypal virulent strain (genotype BrI, #6) and a non-archetypal intermediate virulent isolate (genotype BrIII, #8) (Chiebao et al., 2021). In accordance with previous literature, the authors associate a strong and acute Th1 immune response (IFNγ, IL-12 higher levels) with highly lethal strains, whereas a longer and modulatory Th2 immune response was triggered by moderately virulent isolates (TLR-MyD88, CXCR3 expression). Similarly, serum levels of IFNγ, TNF-α, IL-12 p40, IL-10, IL-6, IL-4, and IL-2 were evaluated as virulence markers for the severity of toxoplasmic encephalitis (Araujo and Slifer, 2003).

The cumulative morbidity rate or the severity of clinical signs are additional non-lethal virulence parameters considered in literature. It requires trained personnel, cohesion in the evaluation criteria and, at best, a normalized clinical scoring criterion (Sánchez-Sánchez et al., 2019; Fernández-Escobar et al., 2020; Chiebao et al., 2021; Fernández-Escobar et al., 2021; Pena et al., 2021; Salman et al., 2021). The main clinical signs associated with *T. gondii* infections in mice are (in increasing order of severity) piloerection (ruffled coat), ascites, loss of body weight/condition, prostration, dyspnoea, and neurological signs such as motor incoordination, head tilting, or circling motion. The cumulative morbidity could be calculated based on the number of mice that present any clinical sign after inoculation divided by the number of infected mice (Fernández-Escobar et al., 2020). Another strategy to quantify such a subjective aspect is the representation of the daily scoring mean value variation and standard deviation according to the clinical findings; both aspects were successfully evaluated for mice experimentally infected with type II (#3), BrI (#9) or BrIII (#8) genotypes elsewhere (Chiebao et al., 2021).

In some Danish studies, levels of the acute-phase reactant haptoglobin in serum were considered for evaluation as an additional virulence parameter along mice infection with *T. gondii* (Jensen et al., 1998; Jungersen et al., 1999; Jungersen et al., 2002). Apparently, strains that caused more severe body weight loss also induced the highest serum haptoglobin and specific anti-*T. gondii* antibodies concentrations during the acute phase of the infection. Weight loss and anti-*T. gondii* IgG response have been used in other studies although, similarly to haptoglobin levels, not extensively, probably due to the excessive handling of animals required and a weak correlation with mortality rates (Kannan et al., 2010; Bezerra et al., 2019; Masatani et al., 2020; Salman et al., 2021; Yang et al., 2021). Interestingly, some studies also approached the analysis of behavioral changes in mice infected by different *T. gondii* reference strains, through the evaluation of learning and memory, locomotor activity, spatial working and aversion to feline odour, among other parameters (Kannan et al., 2010; Bezerra et al., 2019); however, such complex assessments require special devices to monitor mice activity. The study of behavioral changes as phenotypic markers associated with infection could be of special interest since the relationship between toxoplasmosis and human neurological disorders and psychiatric illnesses such as schizophrenia and bipolar disorder has been repeatedly demonstrated (Xiao et al., 2013; Chaudhury and Ramana, 2019; Wang et al., 2019).

Despite attempts to describe and incorporate new non-lethal virulence parameters, the main problem we face is the lack of homogeneity in the selection and in the experimental conditions between studies. Time points of infection, procedures and analytical methods vary completely between different investigations (**Supplementary Table S1**). However, despite this lack of consensus protocols, it is undeniable that there is a growing interest, as shown within the literature, in going beyond the mere mortality rate calculation in the assessment of virulence for different *T. gondii* strains using the mouse model.

An additional approach to virulence degree prediction can be achieved based on molecular analyses. The *CS3* locus has been described previously as a highly predictive marker of mortality in mice challenged with *T. gondii* isolates (Pena et al., 2008); high mortality rates associated with the type I or II alleles of the *CS3* region, and low or null rates associated with the type III alleles have been reported (Pena et al., 2008; Wang et al., 2013b; Rocha et al., 2018). However, several subsequent findings contradicted such observations (Langoni et al., 2012; Rêgo et al., 2017; Fernández-Escobar et al., 2020). Currently, *ROP18* and *ROP5* are well-known virulence factors in *Toxoplasma* virulence in mice (Behnke et al., 2015; Xia et al., 2021), and their allelic combination was proposed as highly predictive of virulence in mice across globally distributed *T. gondii* isolates (Dubey et al., 2014; Shwab et al., 2016). As detailed in above studies, some allelic combination such as 2/2 or 4/4 has been found firmly associated with certain mortality degrees (0% or 100% lethality, respectively); however, other genetic combinations such as 3/1 or 4/3 are much less predictive, and ultimately, the combination 3/3 is the most unspecific profile due to its association with levels of mortality strongly varying from 100 to 0% (Shwab et al., 2016; Hamilton et al., 2019; Uzelac et al., 2020; Bernstein et al., 2021; Fernández-Escobar et al., 2021). In summary, although molecular tools seem useful to predict the virulence degree to some extent, additional genetic factors might be also involved. It should be taken into account that despite the large database used for these correlations at a global level (Shwab et al., 2016), with up to 240 records, it included mouse mortality data calculated at the time of strain isolation by bioassay in mice. In the light of gathered data (**Supplementary Table S1**), a re-assessment of genotype-phenotype correlation, considering only mice mortality data obtained from standardised mouse infection models, could yield more reliable results. A continuous collection of *ROP18* and *ROP5* allelic types and mouse virulence data will facilitate future studies to identify other virulence genes, and ultimately improve the prediction power of genotyping for virulence.

### *In Vitro* Models

The current animal welfare policies not only highlight the necessity to refine the *in vivo* procedures but also to minimize and replace the use of laboratory animals. In this context, the use of *in vitro* models represents an excellent alternative for the study of intracellular organisms such as *T. gondii.* The *in vitro* models allow the study of the host cell infection process by the tachyzoite stage, namely the lytic cycle, which mimics the dissemination of the parasite during the acute phase of the infection (Black and Boothroyd, 2000). The lytic cycle of *T. gondii* is a tightly regulated process, which includes adhesion to the host cell, invasion, PV formation, multiplication, and egress steps (Sibley, 2010). It is important to note that *in vitro* experiments reflect the behavior of the parasite in the absence of selective pressures during an infection of a host, which explains that the results may easily not correspond to what occurs in an *in vivo* assay. In the case of *T. gondii*, given its enormous plasticity represented in a vast host range, *in vitro* experiments can provide a less biased, host-centered view, although it will always be necessary to contextualize the results (Poulin and Combes, 1999).

Proliferative stages of the parasite have been cultured *in vitro* employing a variety of cell culture lines (*e.g.*, HeLa, Vero, HFF, BeWo) and primary cell cultures (Scheidegger et al., 2005; Müller and Hemphill, 2013), and among them, target cells or tissues (*e.g.*, trophoblast and nervous cells, dendritic cells [DCs] or macrophages) should be highlighted (Guimarães et al., 2008; Dellacasa-Lindberg et al., 2011; Mammari et al., 2014; Witola et al., 2014; Barbosa et al., 2015; da Silva et al., 2017; Pacheco et al., 2020). Most of publications on *T. gondii* implementing *in vitro* assays are focused on safety and efficacy assessment of potential antiparasitic drugs (Basto et al., 2017; Murata et al., 2017; Radke et al., 2018) or on demonstrating the role of different host and parasite effectors in the *T. gondii* lytic cycle (Camejo et al., 2014; Bai et al., 2018; Guo et al., 2019; Wang et al., 2020). *In vitro* models are considered also suitable first approaches to phenotypically characterize apicomplexan parasite strains (Regidor-Cerrillo et al., 2011; Dellarupe et al., 2014; Frey et al., 2016; Jiménez-Pelayo et al., 2017; García-Sánchez et al., 2019). However, only a small proportion of the publications addresses the virulence characterization of non-laboratory *T. gondii* isolates *in vitro* (Loeuillet et al., 2019; Sánchez-Sánchez et al., 2019; Bernstein et al., 2020; Uzelac et al., 2020; Fernández-Escobar et al., 2021; Salman et al., 2021).

A systematic review of literature (Contreras-Ochoa et al., 2012) compared studies that had used mouse and human glial cell cultures to determine *T. gondii* invasion and replication rates in these cells. The wide experimental heterogeneity found hampers drawing definitive conclusions but type II strains (ME49 [#1] and PRU [#3]) seem to be less invasive of nervous system-derived cells than type I (RH [#10] and BK [genotype # unknown]) strains. Several publications have characterized some *in vitro* virulence parameters of the *par excellence T. gondii* strain RH in different cell types, and it has been extensively used as an experimental control. However, the RH strain has been maintained and passed through lab mice or cell culture for several decades and its biological behaviour has drastically changed (Khan et al., 2009). Comparative studies of other different laboratory strains have been also published (Appleford and Smith, 1997; Diana et al., 2004; Fux et al., 2007; Lambert et al., 2009; Cañedo-Solares et al., 2013; Mammari et al., 2014). Overall, it is generally claimed that type I strains present enhanced proliferation capacity and lower host immune system stimulation than type II isolates (Mammari et al., 2014). Regarding the *in vitro* virulence assessment of non-laboratory (field) isolates, the number of studies is relatively low; however, its use as a complement to the evaluation of virulence in mice has increased in recent years (Loeuillet et al., 2019; Sánchez-Sánchez et al., 2019; Bernstein et al., 2020; Fukumoto et al., 2020; Uzelac et al., 2020; Fernández-Escobar et al., 2021; Salman et al., 2021).

Reviewing the *T. gondii* literature revealed that *in vitro* phenotypic evaluation is mostly based on parameters such as parasite invasion rate, proliferation kinetics, tachyzoite yield (TY), or on the assessment of plaque formation, tachyzoite-bradyzoite conversion and spontaneous cyst-formation (Regidor-Cerrillo et al., 2011; Li et al., 2014; Loeuillet et al., 2019; Sánchez-Sánchez et al., 2019; Uzelac et al., 2020; Fernández-Escobar et al., 2021; Salman et al., 2021). Data on the use of the main virulence parameters in recent remarkable investigations are summarized in **Table 2**. Other interesting but less extended *in vitro* parameters can be found in the literature. For example, in a Chinese study (Zhang et al., 2013) activation and polarization of macrophages after infection with a highly virulent or a mildly virulent ToxoDB genotype #9 strains were studied in primary BMMφs and peritoneal Mφs from mice and Raw 264.7 cells (mouse macrophages), showing completely opposite phenotypes. In a complex study (Barragan and Sibley, 2002), *in vitro* migration of 20 *T. gondii* strains was measured using a transwell system based on polarized Madin-Darby canine kidney (MDCK) and human foreskin fibroblast (HFF) cell monolayers co-cultures, as well as in HFF cell monolayers covered with agarose. The transwell system consisted of the upper chamber with polarized MDCK and HFF cell monolayer on a filter and the lower chamber with a HFF cell monolayer. Freshly released parasites were added to the upper chamber of the transwell system and migration of parasites to the lower chamber HFF monolayer was quantified. Both approaches revealed a superior migratory capacity of Type I overtype II and type III strains. These *in vitro* assays need to be tested with more *T. gondii* strains to confirm their significance in predicting parasite virulence in the future.

**Table 2:** Summary of *in vitro* suitable parameters for the virulence evaluation of *Toxoplasma gondii*.

Parameters evaluated	Cell line	Experimental conditions	Method	Calculation	Reference
**Invasion rate**	AH-1 (ovine trophoblast)	200 tachyzoites infecting 2 × 10^5^ cells for 4, 8 or 56 hpi	Immunofluorescence staining at 56 hpi	Number of infection events observed divided by two to estimate the percentage of invading tachyzoites	Fernández-Escobar et al., 2021
	Vero	MOI 1:1 for 1 h	Immunofluorescence staining at 6 hpi	Total number of PVs observed	Bernstein et al., 2020
	HFF	NA	Double immunofluorescence staining	Total number of cell-associated parasites observed, scored as being outside *vs.* inside the cells	Fukumoto et al., 2020
	Vero	MOI 0.1:1	Direct LM observation from 1 to 3 hpi	Counting presence/absence of infected cells	Uzelac et al., 2020
	HFF	NA	Microscopic direct observation of intracellular parasites after Diff Quick coloration at 1 hpi	Number of infected cells/1,000 cells	Brenier-Pinchart et al., 2010
	HFF	Tachyzoites inoculated at MOI 3:1 for 2, 8, 16, 24 and 48 h	Wright Giemsa staining	Infected and uninfected cells were counted by LM in 20 visual fields (1000 ×). The cell infection rate (%) = number of infected cells/total number of cells × 100	Li et al., 2014
**Proliferation rate**	HFF	Tachyzoites inoculated in confluent cultures at MOI 1:1	Immunofluorescence staining at 0, 24 and 48 hpi	The mean number of parasites/PV	Salman et al., 2021
	Primary rat peritoneal macrophages	MOI 3:1 for 1 h	3H-uracil uptake measurement at 40 hpi	Rate of uracil incorporation evaluation by radioactivity measure	Loeuillet et al., 2019
	AH-1 (ovine trophoblast)	MOI 4:1 for 8 h	Quantification of parasite genomic DNA by qPCR at 8, 24, 32, 48, 56, 72, 80 and 96 hpi	Tachyzoites/ng of total DNA reached at the different time points	Fernández-Escobar et al., 2021
	Vero	Tachyzoites inoculated into 80% confluent cultures at MOI 1:1 for 1 h	Immunofluorescence staining at 18 hpi	Number of PVs with 2 ≥ tachyzoites observed	Bernstein et al., 2020
	HFF	10^5^ tachyzoites inoculated in confluent cultures for 48 h	Quantification of parasite genomic DNA by qPCR	Tachyzoite yield (number of tachyzoites produced) at 48 hpi	Sánchez-Sánchez et al., 2019
	Vero	MOI 0.1:1	Direct LM counting of parasites inside PV at 48 hpi	Number of divisions was estimated as log2 of the number of parasites per PV	Uzelac et al., 2020
	HFF	2.5 × 10^5^ tachyzoites inoculated for 24 h	3H-uracil uptake measurement	Uracil incorporation evaluation by radioactivity measure	Brenier-Pinchart et al., 2010
	HFF	Tachyzoites inoculated at MOI 3:1 for 2, 8, 16, 24 and 48 h	Wright Giemsa staining	The number of infected cells and tachyzoites inside cells was counted by LM in 20 visual fields (1000 ×). The mean of tachyzoites per infection cell = total number of tachyzoites in infected cells/total number of infected cells	Li et al., 2014
	Sarcoma 180	Tachyzoites inoculated in confluent cultures (culture flasks) at MOI 3:1	Cell suspension daily collection, from 1 to 6 dpi. Tachyzoites were counted in a hemocytometer and their viability determined by the trypan blue exclusion test	Fold-change calculation	Chai et al., 2003
**Cyst formation capability**	MARC-145	2 × 10^3^ tachyzoites inoculated in confluent cultures (24-well plates) for 8 hpi. Conversion induction by culture medium at pH 8 for 4 days	Double immunostaining (DBL and anti-*T. gondii* antibodies)	Direct LM counting of PVs, lysis plates (DBL-negative) or tissue cyst (DBL-positive)	Ribeiro-Andrade et al., 2019
	HFF	Tachyzoites inoculated in confluent cultures at MOI 1:1	Double immunostaining (DBL and BAG1)	Direct LM observation (presence/absence)	Salman et al., 2021
	Primary mouse peritoneal macrophages	Tachyzoites inoculated in confluent cultures at MOI 0.25:1	Double immunostaining (DBL and BAG1)	Direct LM observation (presence/absence)	
	MARC-145	2 × 10^3^ tachyzoites inoculated in confluent cultures for 24 h. Conversion induction by culture medium at pH 8–8.2 for 3-4 days	Immunostaining (DBL)	Direct LM observation. DBL-positive and DBL-negative parasite structures rate	Sánchez-Sánchez et al., 2019
	HFF	1 × 10^4^ tachyzoites inoculated in confluent cultures for 5 days. Conversion induction by culture medium at pH 8.1 for 7 days	Immunostaining (DBA)	Direct LM observation (presence/absence)	Fukumoto et al., 2020
	HFF	10^4^ parasites inoculated for 7 days. Cells were treated or not with 500 U/ml of IFN-γ on 0 and 4 dpi	Immunostaining (BSR4)	Direct LM counting	Brenier-Pinchart et al., 2010
	HFF	Tachyzoites inoculated in confluent cultures (6-well plates) for 3 to 9 days Conversion induction by culture medium at pH 8.1	Immunostaining (DBL, BAG1)	Direct LM observation. Average number of cysts per 40X magnification field and average cyst sizes (area in µm^2^) were calculated	Fux et al., 2007
**Plaque formation**	Vero	5 × 10^4^ tachyzoites inoculated in confluent cultures for 4 days	0.2% crystal violet solution in 2% ethanol staining	Direct LM observation	Sánchez-Sánchez et al., 2019
	Vero	Tachyzoites inoculated in confluent cultures at MOI 0.5 for 10 days	1% crystal violet solution staining	Direct LM observation and counting	Uzelac et al., 2020

HFF, human foreskin fibroblasts; hpi, hours post-infection; MOI, multiplicity of infection; PV, parasitophorous vacuole; NA, not available; LM, light microscopy; DBL, Dolichos biflorus lectin; DBA, Dolichos biflorus agglutinin; BAG1, bradyzoite antigen 1; BSR4, bradyzoite surface antigen.

One of the key issues is that there is no consensus in experimental conditions (*e.g.*, multiplicity of infection [MOI], number of passages, time points for infection, cell culture lines, methods of analysis, among others), yielding non-comparable results (**Table 2**) (Contreras-Ochoa et al., 2012).

### *Ex Vivo* Models

*In vitro* assays offer only a partial view of the processes triggered during infection, since the interaction between the different cell types that shape an organ or the immune system response, for example, are not reflected. Meanwhile, *ex vivo* models that preserve the cellular architecture and function of certain organs (*e.g.*, placenta) during the *in vitro* study period might be an interesting alternative (Fry et al., 2019). Noteworthy studies on the proliferation of *T. gondii* in explants and organoid-derived monolayers have been carried out (Scheidegger et al., 2005; Robbins et al., 2012; Ander et al., 2018; Holthaus et al., 2021). Practical applications of placental explants in the study of host (human)-parasite (*T. gondii*) interactions *ex vivo* were reviewed recently (Pastor-Fernández et al., 2021), but until now no study has been focused on virulence evaluation of *T. gondii* strains using *ex vivo* approaches. An investigation that deserves attention was carried out by Robbins et al. (2012), in which the preferential infection of different structural components of the placenta and the proliferation capacity in a human placental explant model were evaluated for three reference strains of *T. gondii* of different genetic types (I, II, and III); nevertheless, no statistical significance was observed.

## PROPOSED METHODOLOGY FOR VIRULENCE ASSESSMENT OF *Toxoplasma gondii*

**Figure 2:**
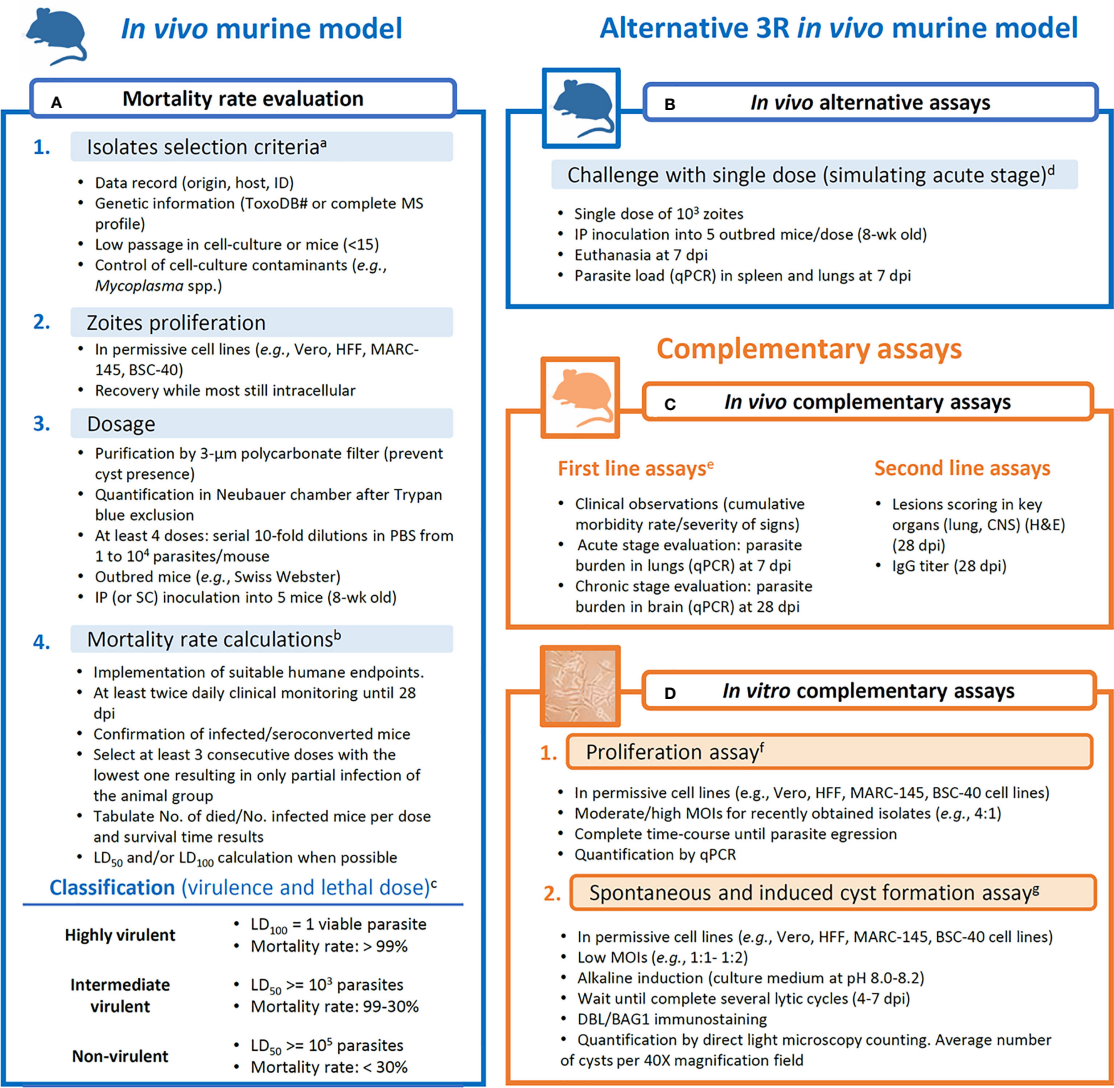
Proposed workflow for assays aiming at obtaining data for the evaluation of the virulence degree of *Toxoplasma gondii* strains. **(A)** Mortality rate calculation is based on Saraf et al. (2017), with complementary analysis like lethal dose calculations and survival time reports. **(B)** Alternative assays to the mortality rate evaluation. **(C)** Complementary *in vivo* assays are divided into first line (more informative) and second line (less informative) parameters/procedures. **(D)** *In vitro* assays are proposed as reliable complementary procedures that limit the inter-host variability. ^a^ *ROP18/5* allele combination and *CS3* profile are considered to have predictive value for the virulence in mice (Pena et al., 2008; Shwab et al., 2016); isolates with low passage history (Khan et al., 2014). ^b^Morbidity scoring and humane endpoint (Pena et al., 2021); tabulation of data (Jiang et al., 2020); LD^50^ calculations (Probit tests). ^c^Virulence classification (Su et al., 2002; Dubey et al., 2014; Saraf et al., 2017). ^d^Parasite tropism and quantification (Hill and Su, 2012; Fernández-Escobar et al., 2020). ^e^Clinical scoring, weight loss, parasite load and histological lesions scoring in additional inoculation groups (10^3^ tachyzoites/mouse) euthanized at 7 or 28 dpi (Fernández-Escobar et al., 2020; Fernández-Escobar et al., 2021). ^f^Proliferation evaluation (Fernández-Escobar et al., 2021). ^g^DBL, *Dolichos biflorus* lectin; spontaneous conversion (Salman et al., 2021); induced conversion (Ribeiro-Andrade et al., 2019); quantification of cysts (Fux et al., 2007).

Given the high heterogeneity within studies observed in the previous sections, it is clear that there is a need to harmonize criteria and methodologies when field strains are being subjected to virulence evaluation. In the present section, taking into consideration bioethical and practical aspects, we aimed to provide an easy-to-follow workflow (**Figure 2**) of reliable assays that may yield repeatable and comparable results.

In **Figure 2A**, an improved and thorough version of the virulence in mice evaluation procedure described by Saraf et al. (2017) is presented. Details on isolates selection criteria or data presentation, among others, are described. As commented earlier, due to national regulations, some animal welfare policies prevent getting ethical approval for the development of mouse assays that include several inoculation groups, and alternatives need to be searched. Although the cumulative mortality approach suggested can provide a base to compare mouse-virulence results from different studies, an alternative to reduce the number of animals in the assays can be solved by selecting an alternative *in vivo* assay (**Figure 2B**). A compromise could be using a single intermediate dose inoculum (10^3^ tachyzoites/animal) and determining parasite load in target tissues such as spleen and lung at 7 dpi. Previous studies showed that *T. gondii* mouse virulence is highly associated with parasite tissue burden, providing an alternative to the cumulative mortality method (Fernández-Escobar et al., 2020, Hill and Su, 2012). From this point, complementary *in vivo* and/or *in vitro* assays are desirable (**Figures 2C**, **D**). Clinical scoring, as well as parasite load and histological lesions scoring determined in key organs have demonstrated to be useful for phenotypic diversity description even between closely related isolates (**Figure 2C**) (Fernández-Escobar et al., 2020; 2021). On the other hand, *in vitro* assays are valuable tools to determine parasite proliferative capacities (proliferation rate and tachyzoite-bradyzoite conversion ability) negating the inter-host variability (**Figure 2D**) (Regidor-Cerrillo et al., 2011; Fernández-Escobar et al., 2021).

## Concluding Remarks

In the present article, a view of the reliable data on the virulence degree of *T. gondii* has been conducted resulting in two main conclusions: first, virulence evaluation is a complex task that should be addressed from multiple approaches, and second, harmonized evaluation criteria and procedures are urgently needed. In addition, an apparent broken linkage between genotype and phenotype have shaken the traditional conceptualization of the virulence degree classification. Here we proposed the baseline for a comprehensive evaluation of *T. gondii* strains virulence by methodologies that should be accessible to most *T. gondii* research laboratories that, if adopted, should result in comparable results between different studies.

Many gaps remain to be solved, and some of these should be taken into consideration when planning further experiments: a) a wider (complete) definition of virulence is necessary and it should combine the study of infection effects on the host and parameters of the parasite’s own fitness; b) in combination with the conventional *in vivo* mouse model, complementary *in vitro* models are valuable strategies to describe host- independent parasite proliferative features; c) and further deep molecular analyses (*e.g.*, whole-genome sequencing and epigenetic studies) are needed to identify new virulence factors involved in the different phenotypes (virulence degrees) observed.

Currently, all approaches need to be planned under the 3Rs (replacement, reduction and refinement) ethical principles and are compulsorily implemented along with strict animal welfare policies (*e.g.*, Regulation [EU] 2019/1010 of the European Parliament and of the Council of 5 June 2019). Furthermore, active searches for non-lethal parameters are of major importance for *in vivo* research to reduce animal suffering. Steps should be taken for increasing the number of isolates evaluated by integrated models since isolates from many geographical areas remain unexplored; a considerable lack of information regarding isolates of human origin is particularly relevant, especially as *T. gondii* is a major zoonotic agent and an excellent example of the One Health concept.

To sum up, through the implementation of integrated methods, a thorough panel of parameters will be available to compare isolates worldwide, and this information will contribute to a risk evaluation assessment for circulating *Toxoplasma* strains in a given area. It is undeniable that interesting future challenges remain for researchers in the field.

## Author Contributions

RC-B, MF-E, and LO-M conceived and designed the manuscript. RC-B, and MF-E extracted data. RC-B, MF-E, FK, CS, and LO-M drafted the manuscript. All authors contributed to the article and approved the submitted version.

## Funding

MF-E is funded by UCM-POP 2021 post-doctoral grants. RC-B, MF-E, and LO-M are part of the TOXOSOURCES consortium supported by the funding from the European Union’s Horizon 2020 Research and Innovation Programme under the grant agreement No. 773830: One Health European Joint Programme. FK is supported by funding from the Scottish Government’s Rural and Environment Science and Analytical Services Division (RESAS) and the Moredun Foundation.

## Conflict of Interest

The authors declare that the research was conducted in the absence of any commercial or financial relationships that could be construed as a potential conflict of interest.

The handling editor JS declared a past co-authorship with the authors CS, MFE, and LOM.

## Publisher’s Note

All claims expressed in this article are solely those of the authors and do not necessarily represent those of their affiliated organizations, or those of the publisher, the editors and the reviewers. Any product that may be evaluated in this article, or claim that may be made by its manufacturer, is not guaranteed or endorsed by the publisher.

## Supplementary Material

Supplementary Table 1 Summary of the available literature reporting virulence evaluation of *Toxoplasma gondii* strains using dosage of tachyzoites in a mouse model.

Supplementary Figure 1 Proportion of *Toxoplasma gondii* isolates subjected to virulence evaluation in a normalized mouse model according to One Health compartment of origin. Note that no isolates obtained from environmental matrices were available. Data used are compiled in **Supplementary Table S1**.
