# Supplementary figures and images for: Unifying Virulence Evaluation in Toxoplasma gondii: A Timely Task
Source: Front Cell Infect Microbiol. 2022 Apr 28;12:868727. doi: 10.3389/fcimb.2022.868727 (PMC9097680; doi:10.3389/fcimb.2022.868727)

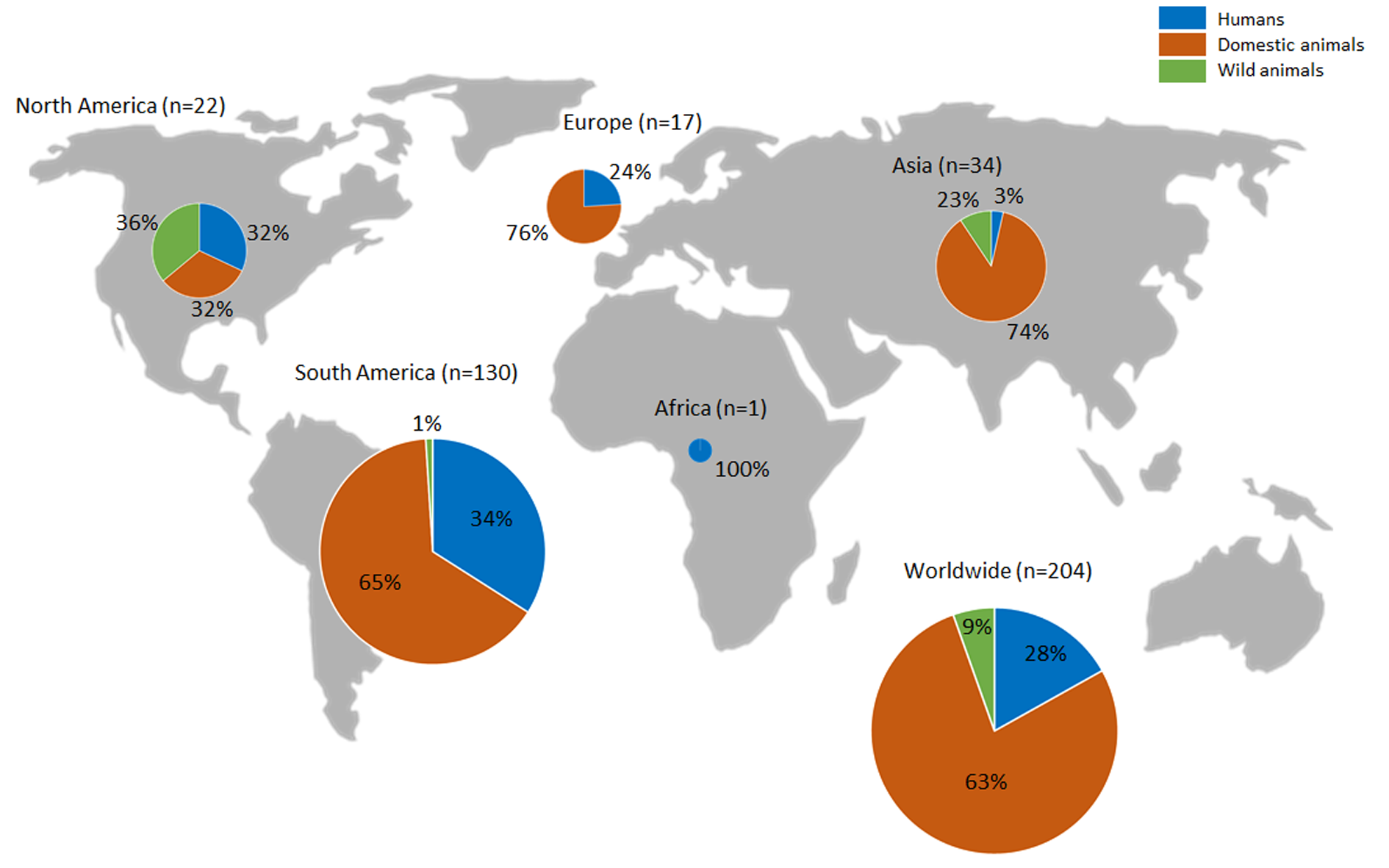

Supplement: Supplementary Figure 1 — Proportion of Toxoplasma gondii isolates subjected to virulence evaluation in a normalized mouse model according to One Health compartment of origin. Note that no isolates obtained from environmental matrices were available. Data used are compiled in Supplementary Table S1 . [file Image_1.tif]
